# Measuring the palpable pulsatility length as a physical examination test in defining the severity of inflow stenosis for hemodialysis fistulas

**DOI:** 10.1186/s12882-019-1536-2

**Published:** 2019-09-13

**Authors:** Matt Chiung-Yu Chen, Mei-Jui Weng, Misoso Yi-Wen Wu, Yi-Chun Liu, Wen-Che Chi

**Affiliations:** 1 Department of Interventional Radiology, Yuan’s General Hospital, No.162, Cheng-gong 1st Rd., Lingya District, Kaohsiung City, 802 Taiwan; 2 Department of Radiology, Kaohsiung Veterans General Hospital, Kaohsiung, Taiwan; 3 Department of Interventional Nursing, Yuan’s General Hospital, Kaohsiung, Taiwan; 4 Department of Nephrology, Yuan’s General Hospital, Kaohsiung, Taiwan

**Keywords:** Hemodialysis, Physical examinations and diagnoses, Arteriovenous shunt, Surgical, Blood flow velocity

## Abstract

**Background:**

Pulsatility is an important property of hemodialysis arteriovenous fistulas (AVF) and can be perceived by the fingers as a gradual decrease in strength downstream from the anastomosis along the main trunk of the fistula. The distance from the point at which the pulse becomes imperceptible to the anastomosis is termed the palpable pulsatility length (PPL); we considered this length may play a role in assessing the severity of inflow stenosis for hemodialysis fistulas.

**Methods:**

This study was performed by retrospective analysis of routinely collected data. Physical examinations and fistula measurements were performed in a selected population of 76 hemodialysis patients with mature fistulas during half a year. Fistula measurements included the PPL before and after treatment and the distance between the anastomosis and the arterial cannulation site (aPump length). The aPump index (API) was calculated by dividing the PPL by the aPump length. Angiograms were reviewed to determine the location and severity of stenosis. PPL and API were used to detect the critical inflow stenosis, which indicates severe inflow stenosis of an AVF.

**Results:**

Receiver operating characteristic analysis showed that the area under the curve was 0.895 for API and 0.878 for PPL. A cutoff value of API < 1.29 and PPL < 11.0 cm were selected to detect the critical inflow stenosis. The sensitivity was 96.0% versus 80.0% and specificity was 84.31% versus 84.31% for API and PPL, respectively.

**Conclusions:**

PPL and API are useful tools in defining the severity of pure inflow stenosis for mature AVFs in the hands of trained examiners with high sensitivity and specificity.

**Electronic supplementary material:**

The online version of this article (10.1186/s12882-019-1536-2) contains supplementary material, which is available to authorized users.

## Background

Pulsatility is an important property of hemodialysis arteriovenous fistulas (AVF) and its strength on palpation can be used to assess the severity of AVF stenosis. Hyperpulsatility indicates the presence of downstream stenosis; while hypopulsatility suggested the presence of inflow stenosis [1]. The amplitude of arterial pulsatility decreases with distance from the heart [2]. An AVF was physiologically an arterialized vein and Huberts et al. [3] mentioned the pulsatility in AVF decreases distally from the anastomosis in newly created AVFs. In our daily practice, we found the length of pulsatility measured from where the pulse becomes not palpable by the finger to the arteriovenous anastomosis (the palpable pulsatility length, PPL) is shorter in those AVFs referred for inflow problems than in those with other complications. In this study, we aim to evaluate the PPL as a diagnostic test for assessing the severity of inflow stenosis and to report its diagnostic accuracy.

## Methods

During the study period, all AVFs were treated and followed up in accord with our routine protocols. From May 2018 to November 2018, 76 patients who had been referred to our institution for treatment of dysfunctional hemodialysis vascular access sites were selected in this study. The inclusion criteria included 1) a mature AVF (after 6 months of use for hemodialysis) with a single main trunk available for cannulation; 2) a mature AVF which was superficial and visible at least from the anastomosis to the arterial cannulation segment. The exclusion criteria included 1) a thrombotic AVF; 2) a sub-optimally treated AVF with a residual stenosis> 30% at the completion of treatment; 3) an arteriovenous graft, 4) an immature fistula; 5) a deformed AVF without a main trunk (the eighth note deformity) [4] or with more than two main trunks (the Gracz’s fistula); or 6) the AVF was too deep to be visible or was difficult to palpate because of interposition graft or heavy calcification.

Before and after each angioplasty treatment of AVF, physical examination (PE) was performed in the angiographic room with the patient supine on the table by a trained vascular access team nurse (M.W) and the operator interventional radiologist (M.C). After one assessor had completed the PE form and left the room, the angiographic nurse allowed the other assessor to perform PE and record the results. During the study period, the PE assessors were unaware of the indication by which the patient was referred for treatment before performing PE. The referral sheet of the patient was kept by the angiographic nurse and was given to the operator interventional radiologist (M.C)“until” the PE form had been completed. Angiography findings were reviewed by a diagnostic radiologist (M.J) who was unaware of the reason for the patient’s referral for treatment or the results of PE.

### Definitions

**Fig. 1:**
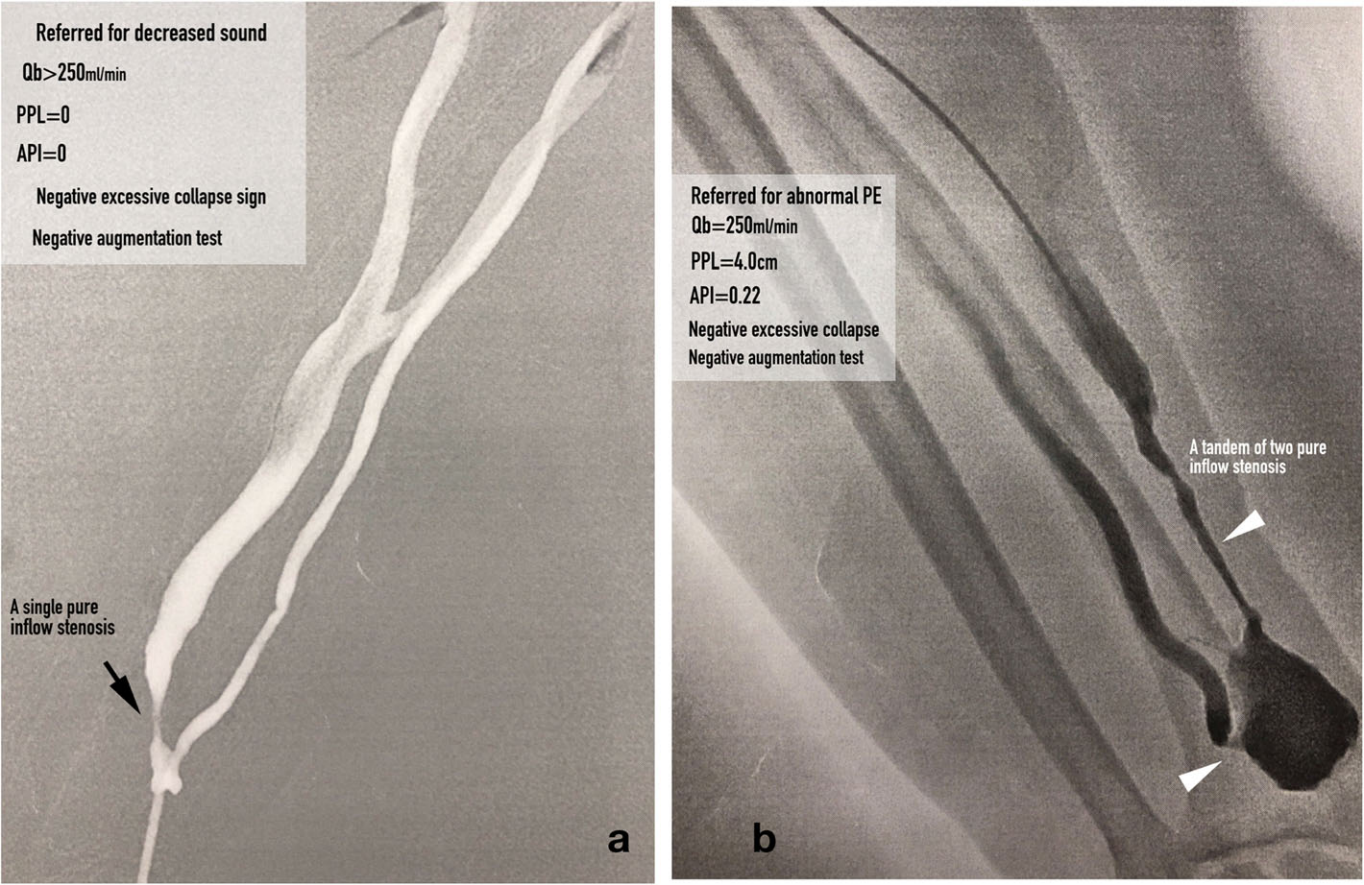
**a** An asymptomatic AVF with a single inflow stenosis, which was diagnosed as a critical inflow stenosis because of PPL and API were both zero (the figure was created by Matt Chiung-Yu Chen). **b** An asymptomatic AVF with a tandem of two inflow stenoses including an anastomotic and a peri-anastomotic stenosis. A critical inflow stenosis was diagnosed because of PPL < 11.0 cm and API < 1.29 (the figure was created by Matt Chiung-Yu Chen)

**An inflow stenosis** was defined as a single stenosis (Fig. 1a) or a tandem of stenoses (Fig. 1b) located in the feeder artery, anastomosis and peri-anastomosis area. Evidence of **inadequate inflow** was defined as 1) blood flow rate (Qb)was < 250 ml/min; and/or 2) AVF suction or tubing shaking during hemodialysis. **A symptomatic inflow stenosis** was defined as an inflow stenosis that resulted in evidence of inadequate inflow. **A critical inflow stenosis** was defined any inflow stenosis that had an equivalent low level of pressure energy as those inflow stenoses that resulted in evidence of inadequate inflow. Such a low level of pressure energy can be assessed by an augmentation test or PPL and API measurements. A critical inflow stenosis can be with or without evidence of inadequate inflow. **A robust AVF** was defined as achievement of full dilation of a culprit stenosis of a dysfunctional AVF with restoration of optimal flow rate and volume in the AVF.

#### The vascular access pump (aPump)

**Fig. 2:**
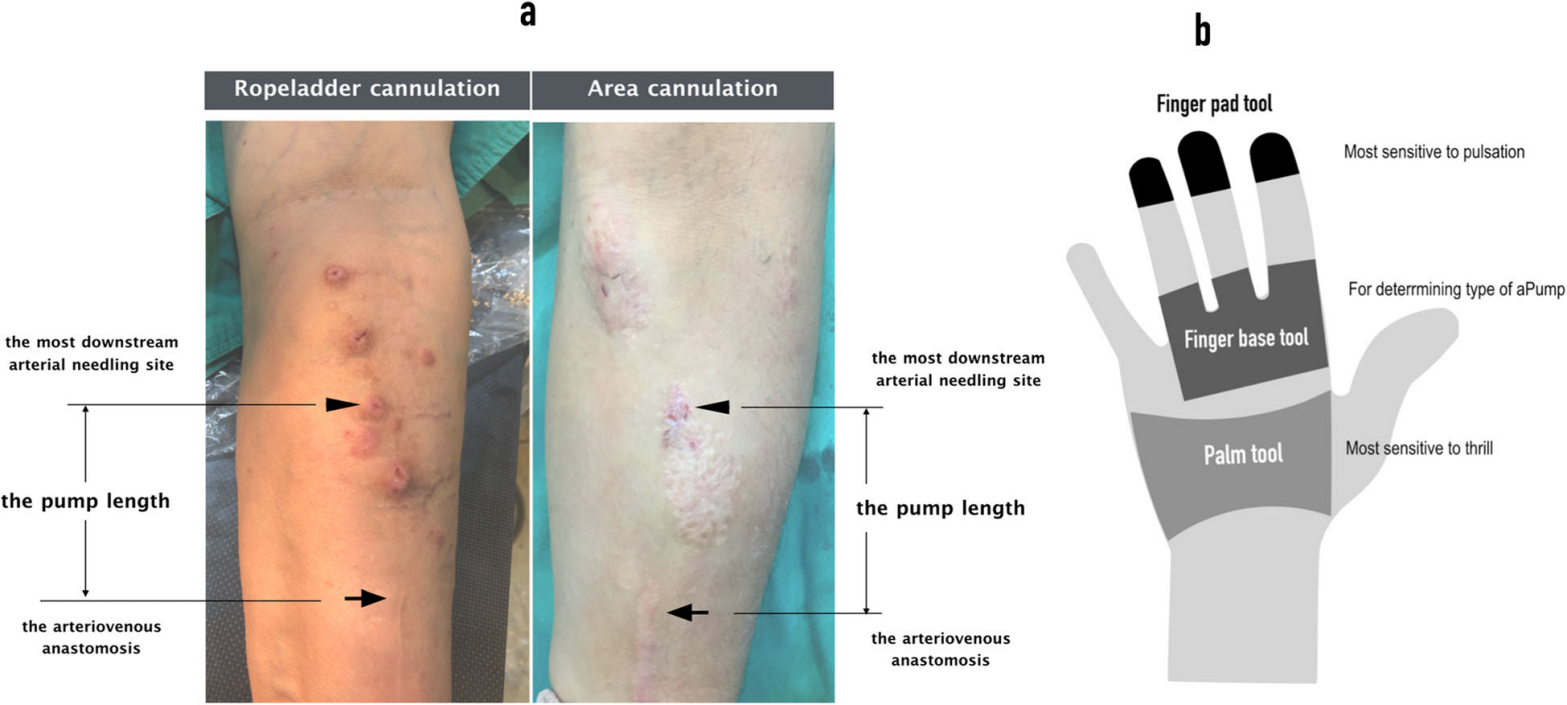
**a** Definition of aPump in AVF cannulated by rope-ladder and area needling techniques (the figure was created by Matt Chiung-Yu Chen). **b** The hand tools for palpating an AVF (the figure was created by Matt Chiung-Yu Chen)

It is not necessary for the whole length of an AVF to be capable of providing a high volumetric flow for hemodialysis. An AVF is usually cannulated around 5–10 cm downstream from the anastomosis (the arterial needling segment). Instead of considering this segment merely as a conduit, we consider this segment of an AVF together with the feeder artery and anastomosis to be a functional structure like the engine of a car and term it as the access pump (aPump), which provides fast volumetric flow for dialysis use. The aPump of an AVF was defined as the segment between the arteriovenous anastomosis (Fig. 2a, black arrow) to the most downstream arterial needling site (Fig. 2a, black arrowhead) and the distance between the two locations as the aPump length (Fig. 2a). An adequate aPump was defined as Qb > =250 mL/min that could support hemodialysis for 4 h at this rate without tubing suction or shaking. Such an adequate aPump reliably provides enough volumetric flow for hemodialysis wherever the arterial needle is inserted along it.

#### Palpable Pulsatility length (PPL)

Because the fingertip is the most sensitive part of the hand to pulsation [1], the pulsatility of an AVF was felt with the fingertip, starting from the anastomosis downward along the main trunk of the AVF to where the pulsation became imperceptible. Following are some tips for measuring PPL (see the Additional file 1: video). 1) Place one index finger around the anastomosis to feel the pulse (the reference pulse), which is usually strong, and then feel the pulse with the other index finger downstream along the AVF (the test pulse) until the test pulse becomes imperceptible in comparison with the reference pulse. 2) Pulsation is usually less obvious around the most pronounced site of the thrill but may reappear if you keep moving downstream. 3) If the test pulse is not easy to detect, as can occur with a deeply seated AVF, compressing the AVF more strongly with the test finger may be helpful. However, if the pulse at the reference finger became stronger as a thrill appears on your test finger, you have probably pressed the AVF too hard with your test finger. 4) If a PPL is long and can be measured to the elbow joint or clavicle, it is considered long enough and measurement is therefore stopped around the joint and the length from anastomosis to the elbow joint or clavicle recorded as its PPL.


Additional file 1:Measuring the palpable pulsatility length (PPL). An animated demonstration of how the PPL was measured in this study. (MP4 2241 kb)


#### aPump index (API)

The adequacy of AVF inflow is affected not only by the presence of inflow stenosis but also the location of insertion of the arterial needle along the aPump. It is not uncommon for a dialysis nurse to attempt a new arterial cannulation site that is a few centimeters closer to the anastomosis and for the pulsation to be stronger than at the initial arterial cannulation site when that site has failed to provide adequate inflow. To minimize the effect of location, the PPL was divided by the aPump length, which was calculated by the formula: aPump index (API) = PPL/aPump length. API was used in this study to diagnose critical inflow stenosis in an AVF.

### Hypothesis 1: lower intra-AVF pressure energy correlate with shorter PPL

The hemodynamic characteristics associated with a stenosis in an artery are 1) pre-stenotic pressure buildup and 2) post-stenotic pressure drop [5, 6]. When pressure waves pass through an inflow stenosis, the pressure energy is dissipated by being converted to kinetic energy and is attenuated downstream to the anastomosis along the length of an AVF according to Poiseuille’s law. Because of this pressure drop, the AVF is under low pressure; thus, the post-stenotic pulsatility of the AVF should decrease and become weak on palpation and the PPL should be short. When a stenosis or a tandem of stenoses is identified only in the inflow segment of an AVF (pure inflow stenosis, Fig. 1a and b), the aPump is considered to be under low pressure and, on physical examination, the AVF is usually felt as a flat access or is poorly augmented during the augmentation test [1, 7, 8]. When a stenosis is identified only in the outflow segment of an AVF (pure outflow stenosis), the aPump is considered to be under high pressure status because of abnormal pressure buildup from the arteriovenous anastomosis to the stenotic site and the PPL should be long. When the outflow stenosis is severe, physical examination usually reveals a strong bounding pulse [1, 8, 9]. We tested this hypothesis by comparing the PPL between AVFs with low pressure aPumps (case group) and high pressure aPumps (control group) in the following three situations:

***Situation 1:*** AVF with pure inflow stenosis (case) versus AVF with pure outflow stenosis (control); ***Situation 2***: AVF with inadequate inflow symptoms (case) versus AVF without inadequate inflow symptoms (control); and ***Situation 3***: Comparisons of PPL before (case) and after (control) percutaneous transluminal angioplasty (PTA) for AVFs in the above-mentioned three situations. Theoretically, after any inflow stenoses have been corrected, the initial low pressure aPump in a dysfunctional AVF would become a higher pressure aPump in a robust AVF. Therefore, the PPL in a robust AVF should be longer than that in a dysfunctional AVF.

**Hypothesis 2:** The severity of an inflow stenosis can be estimated by measuring PPL and API. We hypothesized that the shorter the PPL or the smaller the API value, the more severe the inflow stenosis would be. To characterize low pressure aPumps in AVFs with symptomatic inflow stenosis by measuring PPL and API, a receiver-operator curve (ROC) analysis was performed and Youden’s index used to determine the cut-off values for discrimination of symptomatic inflow stenosis (case group) from all other types of stenosis (control group).

#### Inter-observer agreement for the two assessors

The inter-observer agreement for the two assessors (M.C and M.W) on diagnosis of critical inflow stenosis was assessed by Cohen’s kappa (κ). κ values was used as a measure of the level of agreement beyond chance between the two assessors for PE, those values ranging from 0 to 1.0, with zero indicating no agreement beyond chance and 1.0 denoting perfect agreement. A κ value exceeding 0.80 was considered to denote substantial and a near perfect agreement.

### Statistics

PPL and API in Situations 1 and 2 were compared by unpaired t-test, whereas the comparisons for Situation 3 were performed by paired *t*-test. Calculation of sensitivity, specificity, positive and negative predictive rates for PPL and API in diagnosis of the critical inflow stenosis was performed by the χ^2^ test. Comparisons of PPLs and APIs between groups were performed by one-way ANOVA. ROC analysis and the above-mentioned analyses were performed by Prism Version 6.0 for Mac (GraphPad Software, La Jolla, CA, USA).

## Results

Data on 76 patients (43 male and 33 female) were examined in this study, their mean age being 61.65 ± 2.79 years (range 26–84 years). There were 59 radiocephalic, 11 brachiocephalic, and six brachiobasilic fistulas. The indications for assessment of their fistulas were: 1) inflow problems, including Qb < 250 mL/min (*n* = 25), Qb ≥ 250 mL/min with a Qb reduction by 25% (*n* = 6) and abnormal PE findings including a palpated inflow stenosis with concomitant weak sounds on auscultation (*n* = 8); 2) outflow problems including dynamic venous pressure ≥ 180 mmHg and prolonged bleeding (*n* = 24); 3) difficult cannulation (n = 8); and 4) limb swelling (*n* = 5).

### Hypothesis 1

**Fig. 3:**
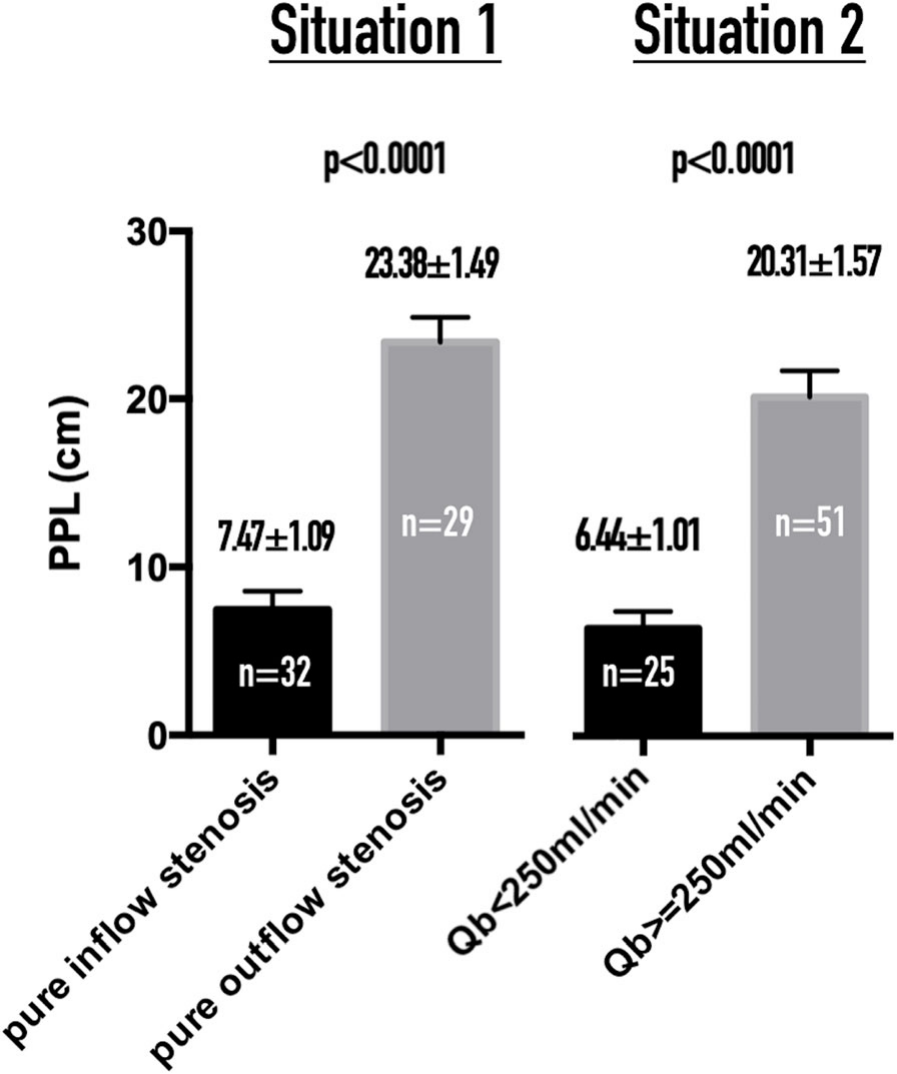
Comparisons of PPL between aPumps in low- and high-pressure status

The PPL in an AVF where the aPump is under low pressure (case group) is significantly shorter than in one under high pressure (control group) for Situations 1 and 2 (*p* < 0.0001; Fig. 3).

**Fig. 4:**
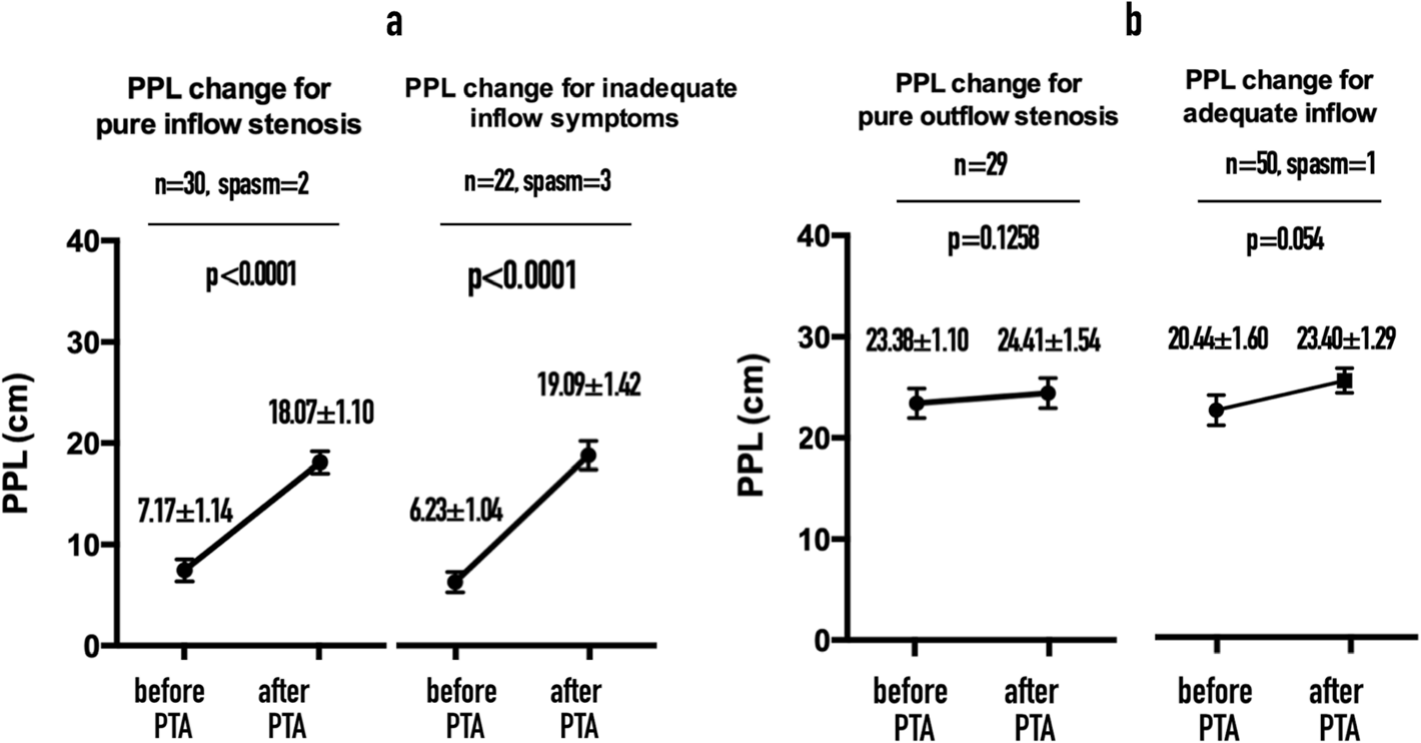
**a** Comparisons of PPL before and after PTA for AVFs in low pressure status. **b** Comparisons of PPL before and after PTA for AVFs in high pressure status

For comparisons of PPLs before and after PTA in Situation 3. Post-PTA arterial spasm occurred in four AVFs (#65, #55, #53, and #24); they were accordingly excluded from the comparisons. Thus 72 pairs of dysfunctional and robust AVFs were compared for significant differences. For AVFs with aPumps under low pressure (case groups of Situations 1 and 2), the PPL was significantly longer after PTA than before PTA (*p* < 0.0001; Fig. 4a). For AVFs with aPump under high pressure (control groups of Situations 1 and 2), the PPL was not significantly longer after PTA than before PTA (*p* > 0.05; Fig. 4b). For robust AVFs, the mean PPL (mean ± SEM) was 22.08 ± 1.02 cm and mean API 2.57 ± 0.21.

#### Hypothesis 2

**Fig. 5:**
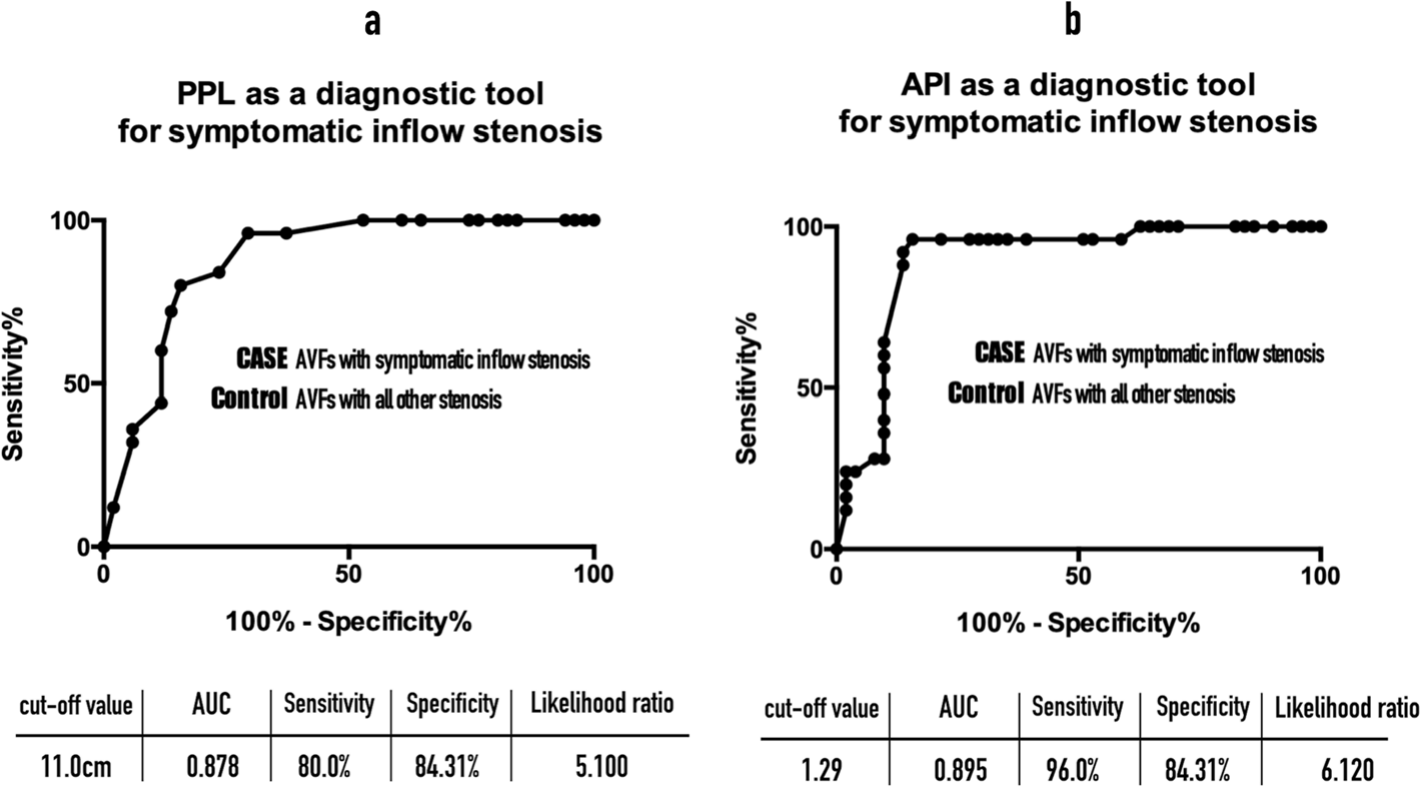
**a** ROC curve for PPL as a diagnostic tool for symptomatic inflow stenosis. **b** ROC curve for API as a diagnostic tool for symptomatic inflow stenosis

Measuring PPL and API enables estimation of the severity of an inflow stenosis and diagnosis of a critical inflow stenosis. To differentiate AVFs with symptomatic inflow stenosis from AVFs with all other types of stenosis, the cut-off value of PPL was 11.0 cm and the AUC was 0.878 (CI 0.802–0.954) with a sensitivity of 80.0% and specificity of 84.31% (Fig. 5a). The API was also found to be an excellent tool for detection of symptomatic inflow stenosis, with a cutoff value of 1.29, AUC 0.895 (CI 0.818–0.972) with a sensitivity of 96.0% and specificity of 84.3% (Fig. 5b). Using PPL < 11.0 cm and/or API < 1.29 as a single diagnostic tool or in combination to detect critical inflow stenosis in AVFs in our patients, the sensitivity, specificity, positive predictive value (PPV) and negative predictive value (NPV) were high, ranging from 71.4–100%, as shown in Table 1.

**Table 1:** The diagnostic accuracies for PPL and/or API in detecting critical inflow stenosis

	Sensitivity	Specificity	Positive predictive value	Negative predictive value
PPL < 11 cm for critical inflow stenosis	83.3%	84.6%	71.4%	91.7%
API < 1.29 for critical inflow stenosis	100.0%	84.6%	75.0%	100.0%
PPL < 11 cm and API < 1.29 for critical inflow stenosis	83.3%	86.5%	74.1%	91.8%
PPL < 11 cm or API < 1.29 for critical inflow stenosis	100%	82.7%	72.7%	100%

#### AVFs with both inflow and outflow stenosis

**Fig. 6:**
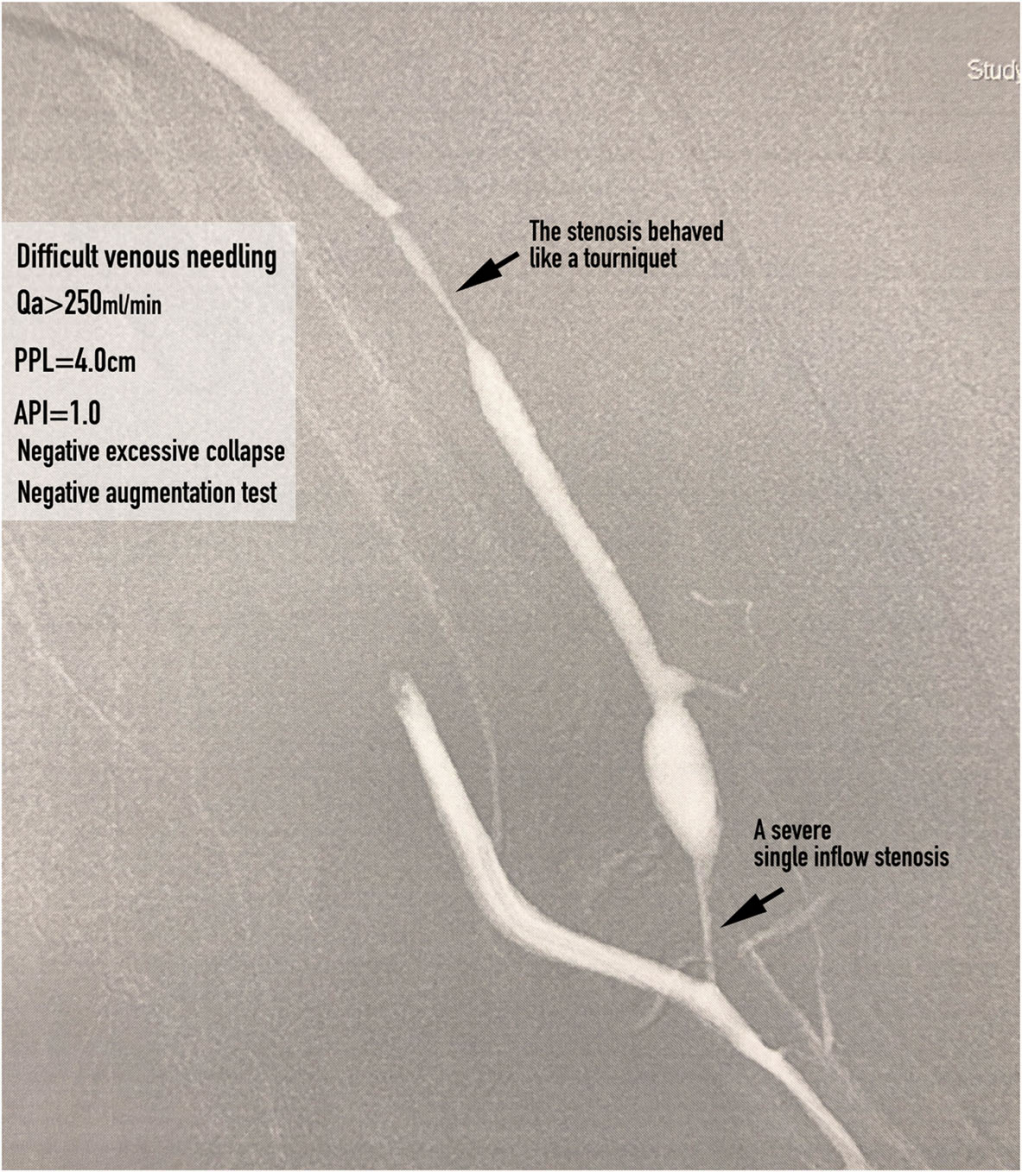
An AVF with both inflow and outflow stenosis (the figure was created by Matt Chiung-Yu Chen)

**Fig. 7:**
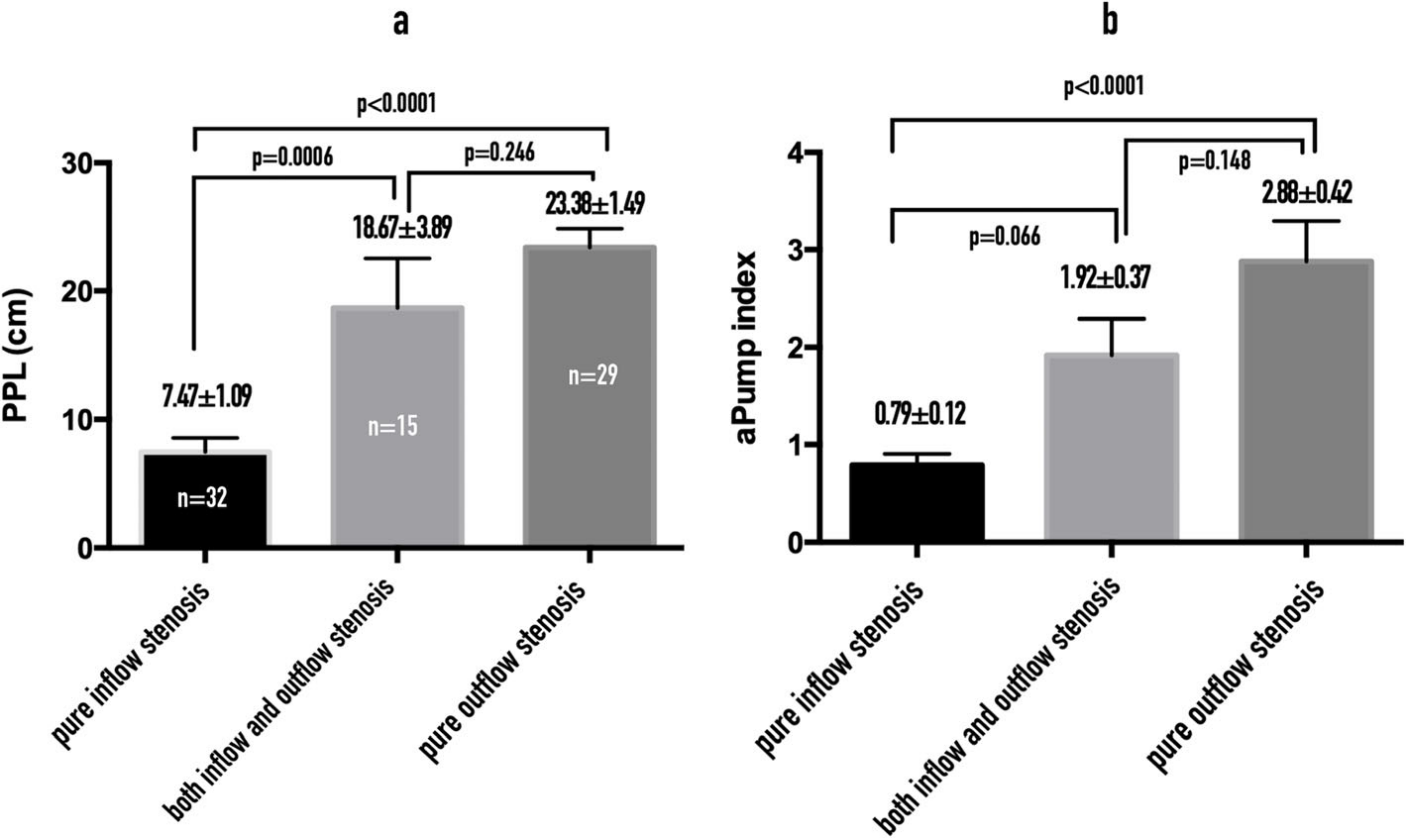
**a** Comparisons of PPL by location of AVF stenosis. **b** Comparisons of API by location of AVF stenosis

**Fig. 8:**
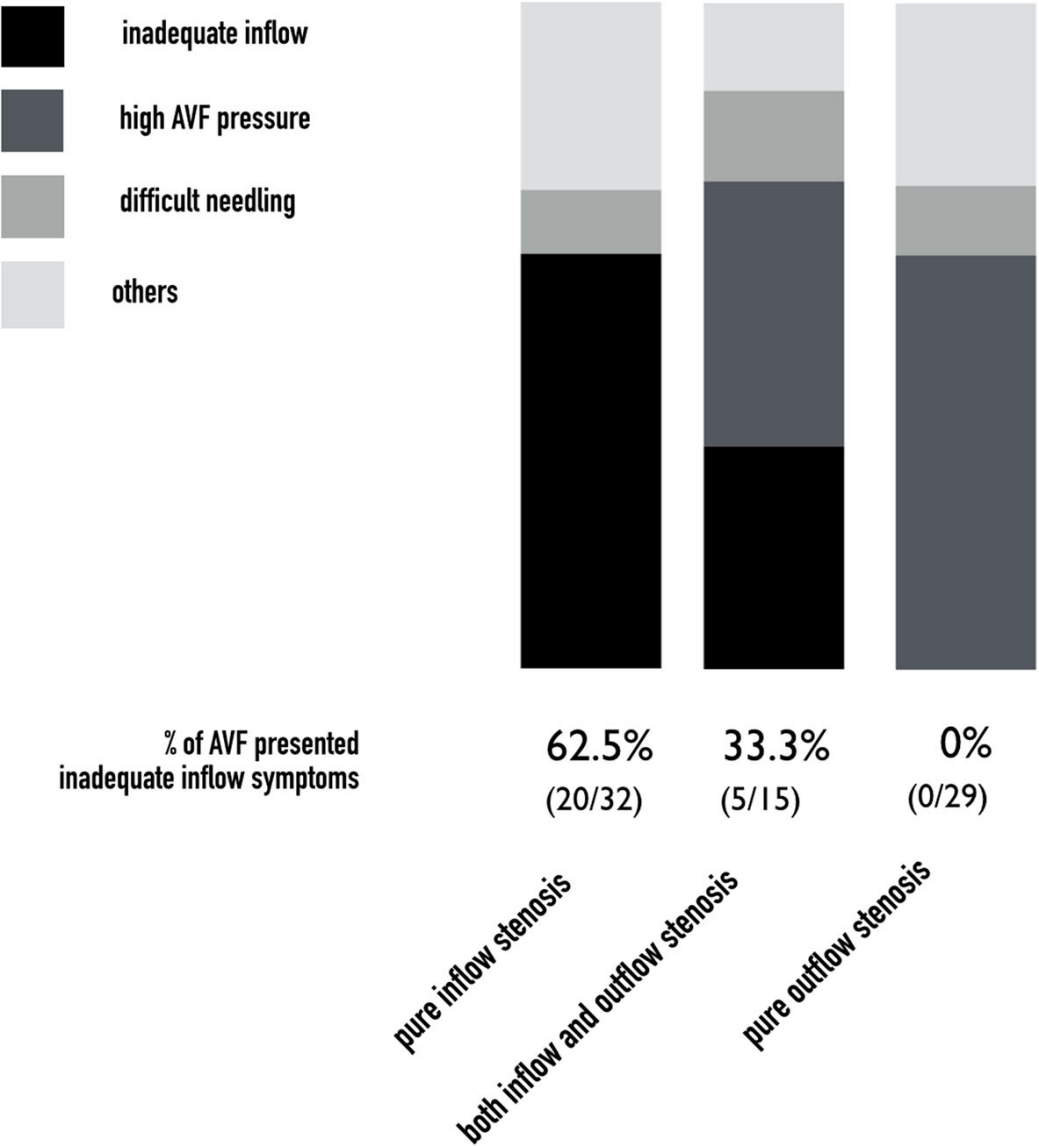
Proportions of inadequate inflow symptoms by locations of AVF stenosis

Comparisons of PPL and API among AVFs with pure inflow stenosis (inflow group), both inflow and outflow stenosis (I/O group; Fig. 6) and pure outflow stenosis (outflow group) were performed by one-way ANOVA (Fig. 7a). The PPL and API for I/O group did not differ significantly from those for the pure outflow group. PPL and API were significantly shorter and smaller, respectively, in the inflow group than in the I/O and outflow groups (Fig. 7b). There were 32 AVFs with pure inflow stenosis, 62.5% (20/32) of which had evidence of inadequate inflow; 15 AVFs with I/O stenosis, 33.3% (5/15) of which had evidence of inadequate inflow; and 29 AVFs with pure outflow stenosis, none of which had evidence of inadequate inflow (Fig. 8).

#### PPL and API in patients with low systemic blood pressure

The blood pressure was measured before PTA and the mean systolic blood pressure at PE was 145.39 ± 6.78 mmHg. Eight patients had systemic blood pressure < 100 mmHg, five of whom were referred for assessment of inadequate inflow of their AVFs and three for Qb < 250 ml/min (*n* = 1) and abnormal PE findings (*n* = 2). The API before PTA was smaller in the hypotension group (1.00 ± 0.68) than in the normal to high blood pressure group (1.90 ± 0.46); however, this difference was not statistically significant (*p* = 0.189). The PPL before PTA was shorter in the hypotension group (9,75 ± 5.89 cm) than that in the normal to high blood pressure group (16.46 ± 2.88 cm); however, this difference was also not statistically significant (*p* = 0.124). PPL and API were not compared before and after PTA because feeder artery spasm occurred in two AVFs, leaving only six pairs of data for the *t*-test.

#### Inter-observer agreement between the two assessors in this study

There was a substantial inter-observer agreement on diagnosis of critical inflow stenosis. For detecting the critical inflow stenosis, the k value was 0.92 by using PPL < 11.0 cm and was 0.89 by API < 1.29.

## Discussion

Our major findings were 1) the PPL was short when there was an inflow stenosis and the aPump was under low pressure and long when there was an outflow or I/O stenosis and the aPump was under high pressure; 2) low pressure in the aPump as a consequence of symptomatic inflow stenosis can be identified by PPL < 11.0 cm and API < 1.29 with a high sensitivity and specificity; and 3) there was a high inter-observer agreement on diagnosis of critical inflow stenosis by PPL < 11.0 cm (k = 0.92) and/or API < 1.29 (k = 0.89).

Repair of stenotic lesions is not recommended merely because they are present [10]. Therefore, the question that needs to be answered is when should a functioning AVF with stenosis detected by palpation in its inflow segment be referred for treatment? The K/DOQI recommends treating hemodynamically and clinically significant stenoses. Hemodynamically significant stenoses in AVFs are currently identified by angiographic analysis and comparison of the percentage narrowing with the adjacent “normal “vessel; however, this method is inaccurate [11]. Given that in daily practice it is not uncommon for an AVF to have more than one > 50% stenosis, how can their actual hemodynamic impact be assessed? Moreover, if there are concomitant collateral veins, their actual hemodynamic impact on the AVF is difficult to determine. Fahrtash et al. [11] proposed an absolute minimal luminal diameter (MLD) of 2.7 mm for defining a hemodynamically significant stenosis in a radio-cephalic AVF. However, it has been reported that the minimal luminal diameter correlated poorly with Qa of a radiocephalic AVF [12]. In our study, we measured PPL and API to assess the consequences of a pressure drop caused by an inflow stenosis. When the measured PPL was < 11.0 cm and/or API < 1.29, which was characteristic of the majority of AVFs with symptomatic inflow stenosis, we considered the inflow stenosis was severe enough to be hemodynamically significant.

We agree with the contention that whether to treat a stenosis should be based on its hemodynamic impact on the AVF rather than the percentage narrowing detected by angiography. The gold standard for assessing the hemodynamic impact of a stenosis is to measure the volumetric flow rate of AVF (Qa) by the ultrasonic dilution technique. Tessitore et al. proposed that the optimal tests for identifying an inflow stenosis are Qa < 650 mL/min combined with a positive PE or Qa < 650 mL/min alone [7]. However, given that Transonic hemodialysis monitors are not widely available in dialysis units, the augmentation test has been the only available tool that have been reported as useful for assessing AVF inflow problems.

In this study, we defined an inflow stenosis incurring a hemodynamical derangement estimated by PPL < 11.0 cm and/or API < 1.29 as a critical inflow stenosis. A symptomatic critical inflow stenosis was of no doubt needed a treatment, however, is it justified to treat an asymptomatic critical inflow stenosis? We analyzed the angiograms of those AVFs with asymptomatic critical inflow stenosis (the false positive results; Figs. 1a, b and 6, we found these asymptomatic critical inflow stenoses had very similar severity of stenosis with those symptomatic ones. Tessitore et al. [13] reported that active blood flow surveillance and pre-emptive repair of subclinical stenosis reduce the thrombosis rate and prolong the functional life of mature AVFs. Therefore, in our opinion, we think it is justifiable to treat asymptomatic critical inflow stenosis and it is currently our routine practice.

We found that not every significant inflow stenosis (> 50%) leads to aPump inadequacy. In this study, we detected evidence of inadequate inflow in only about 1/3 of AVFs with I/O stenosis and we call this phenomenon the “tourniquet effect” (Fig. 6). In our daily practice, it is not uncommon for a dialysis nurse to place a tourniquet around an AVF, as a temporary measure, when the arterial cannulation site is not providing an adequate volumetric flow for hemodialysis but angioplasty was, for example, scheduled one week later because of a long waiting list for our angiographic lab. This usually makes an initially inadequate aPump adequate because the tourniquet creates a pressure buildup between the tourniquet and the arteriovenous anastomosis. Limitations: (1) The study was limited for its retrospective nature. (2) The measurement of PPL is subjective and there could therefore be subject to inter-observer variability. However, in this study the inter-observer agreement was high. (3) PPL and API measurements were performed only in mature AVFs and their clinical application and usefulness in immature AVFs need further validation. (4) Only selected AVFs with a main trunk and a visible and palpable aPump were eligible for PPL measurement, which limited the method’s generalizability. (5) There were only eight patients in this study with a systolic blood pressure less than 100 mmHg; thus, determining the impact of blood pressure on measuring PPL and API needs further study.

## Conclusions

PPL and API are potentially useful tools in defining the severity of pure inflow stenosis for mature AVFs in the hands of trained examiners with high sensitivity and specificity. We consider that treatment of both symptomatic and asymptomatic critical inflow stenoses is justifiable because the magnitude of their post-stenotic pressure energy drops is similar.

## Data Availability

The datasets generated and/ or analyzed during the current the study is available from the corresponding author on reasonable request.

## Abbreviations

API: aPump index
aPump: access pump
AVF: Arteriovenous fistula
PE: Physical examination
PPL: Palpable pulsatility length
ROC: Receiver-operator curve
